# Do Health Professionals Sufficiently Address Patients’ Disposition Toward Changing Their Nutritional and Physical Activity Habits? Findings from a Pilot Study among People with Type 2 Diabetes in Northern Italy

**DOI:** 10.3390/healthcare8040524

**Published:** 2020-12-01

**Authors:** Heike Wieser, Fabio Vittadello, Evi Comploj, Harald Stummer

**Affiliations:** 1 Research Unit, College of Health-Care Professions, 39100 Bolzano, Italy; 2 Medical Informatics and Technology, Department for Public Health, Health Services Research and HTA, UMIT—University for Health Sciences, 6060 Hall in Tyrol, Austria; harald.stummer@umit.at; 3 Explora—Research and Statistical Analysis, 35010 Padova, Italy; fabio.vittadello@centroexplora.it; 4 Scientific Directorate, College of Health-Care Professions, 39100 Bolzano, Italy; evi.comploj@claudiana.bz.it; 5 Department of Urology, Central Hospital of Bolzano, 39100 Bolzano, Italy; 6 Faculty of Business, University Seeburg Castle, 5201 Seekirchen, Austria

**Keywords:** type 2 diabetes mellitus, survey, nutritional and physical activity habits, communication and interaction, health professions, transtheoretical model, stages of change

## Abstract

The aim of our study was to evaluate the disposition of individuals with type 2 diabetes mellitus (DM2) toward changing their nutritional and physical activity habits and associated factors—particularly their perceptions about interacting and communicating with four health professions. Working with a local patients’ association, we invited 364 individuals with DM2, all at least 18 years old, to complete a paper-based survey with questions addressing their experiences of interacting and communicating with general practitioners, nurses, dieticians and diabetologists and about their readiness to change targeted habits, their health literacy and their clinical status. Of the 109 questionnaires collected, 100 were eligible for descriptive and inferential statistical analysis. Regarding nutritional habits, the highest percentage of participants were at the maintenance stage (26%), whereas regarding physical activity habits the highest percentage of participants were at the preparation stage (31%). Significant differences between the habits emerged for four of the five stages and for two psychological processes. The precontemplation stage was most associated with communication-related variables, whereas the maintenance stage was associated with higher health literacy for both habits, and waist-to-height ratio was associated with several stages of change and psychological processes for physical activity habits. Considering aggregated stages (i.e., active or passive stage), significant differences were observed for all psychological processes except readiness to change nutritional habits. Logistic regression analysis revealed associations of the active stage with higher self-efficacy and lower discrepancy processes for both habits. Nutritional habits were associated with normal HbA1c values and physical activity habits with high cholesterol. Understanding the combination of the stages of change and how they relate to psychological processes can afford meaningful insights into the potential internal and external communication skills of health professions and should be examined as possible elements for a patient evaluation model.

## 1. Introduction

National and international associations and guidelines for caring for patients with type 2 diabetes mellitus (DM2) recognize the potential of and need for lifestyle and behavior changes as part of managing the disease [1,2,3,4]. The need for greater focus on addressing processes of care in light of patients’ needs are also supported by various clinical trials and systematic reviews. Researchers in the secondary analysis of the Look AHEAD clinical trial, for example, have concluded that improving the selection of individuals who need significant weight loss and sustaining their progress in losing weight could make a difference [5,6,7,8]. However, too little support seems to be provided by health professionals toward changing the nutritional and physical activity habits of patients with DM2, and their communication remains scarce and insufficiently centered on patients’ needs [9,10,11,12].

In their article “Why is changing health-related behaviour so difficult?” Kelly et al. [13] have listed common errors made by policymakers and even by doctors and other health care professions. Perhaps the greatest error is continuing to assume that providing people with information and talking to them about the negative consequences of their habits will drive them to change their behavior in their daily routines. In turn, those authors have argued for rethinking the mode of working with individuals and the public by allowing patients to make decisions affecting their care and by motivating and empowering them to identify changes in their behavior that they want to prioritize.

As described in Davis et al.’s [14] scoping review, several theories and models about changing health behavior are relevant in public health interventions. In our study, we applied one of the four most used theories of behavior change, Prochaska and DiClemente’s transtheoretical model (TTM) of change, often called “the stage of change model” because it unites several psychological theories to form a circular model of change in which relapse is part of the change process.

In developing the TTM, Prochaska and DiClemente were initially interested in why some people succeed in changing their behavior for better health outcomes, which strategies and processes of change they apply and how, and why others struggle to make progress, if any [15,16,17]. According to their model, people become ready to change their behavior over the course of five stages. First, in the precontemplation stage, people are unaware of their behavioral problem. Next, in the contemplation stage, they realize that they may have a problem and should consider changing their behaviors. Third, in the preparation stage, they decide to change their habits soon (e.g., in the next month), and by the fourth stage, the action stage, they have already made some changes to their behavior in the previous month. Fifth and last, in the maintenance stage, people have practiced the changed behavior for 6 months [18,19,20]. The TTM not only implies an incremental process of change over a series of stages but is also as associated with 8 to 10 other processes of change (e.g., consciousness raising, environmental and self-re-evaluation, dramatic relief, counterconditioning, reinforcement management, helping relationships and stimulus control). Applying those processes, described as actions performed more or less consciously during behavior change, as possible sites for therapeutic interventions [21], seems to form a basis for making progress in the overall process of change. To examine and depict the complexity of change proposed in the model, authors have also described the processes as being associated with other psychological processes, including discrepancy, self-efficacy and temptation [22,23].

Several studies have involved applying the TTM to study self-management abilities such as maintaining a healthy diet and engaging in regular physical activity. In their systematic review, Arafat et al. [24] concluded that the TTM is an effective framework for enhancing the self-management skills of people with DM2. More specifically, Kirk et al. [25] and Guicciardi et al. [26] applied the TTM to examine the physical activity habits of older adults with DM2. Along with Ataee et al. [27], they concluded that self-efficacy is a valid predictor of adopting habits of regular physical activity. Nagaraju et al. [28] found that apart from the stage-based application of processes of change, decisional balance especially influenced patients with DM2 to exercise more regularly.

Beyond that, Centis et al. [29] developed and applied an instrument specifically designed to measure readiness to change dietary and physical activity habits in a group of patients with DM2 at 14 tertiary care centers in Italy. The instrument is based on not only Prochaska et al.’s [17] TTM but also on the motivational interviewing principles developed by Rollnick and Miller [30,31], applied by the authors to measure psychological processes (e.g., discrepancy (weighing the pro and cons of change), importance, self-efficacy (individuals’ evaluation of being able to do the change), temptation (individuals’ feeling of being overwhelmed by the not well enough changed behavior), readiness to change, and stability of change) with visual analog scales and an instrument validated by Spiller et al. [32].

According to Kelly et al. [13], the single stages of change and psychological processes measured in Centis et al.’s [29] study could be influenced by patients’ interaction and communication with health professionals. However, the literature to date contains no work explicitly focused on TTM and patients’ communication and interaction with health care workers in different health professions as possible facilitators of behavior change. In response, we sought to identify what individuals with DM2 report about their disposition and readiness to change their nutritional and physical activity habits, as well as to pinpoint which factors influence their attitude toward and commitment to that process of change.

The aims of our pilot study were to evaluate the disposition and readiness to change nutritional and physical activity habits in a convenience sample of patients with DM2 and to explore which factors are associated with them. In particular, we evaluated how often the patients interacted or communicated with professionals in four health professions and how they perceived those experiences. Ultimately, we wanted to determine how such patient–professional relations and perceptions among patients are associated with the patients’ disposition toward changing their habits.

## 2. Material and Methods

### 2.1. Population

All individuals with DM2 who were at least 18 years old and belonged to a local diabetes association in northern Italy were invited to participate (*N* = 364). Nearly half (48%) of them were males, and 34% were less than 70 years old. Because the population was bilingual, the questionnaire was sent to all participants in their preferred language (i.e., German or Italian).

### 2.2. Data Collection and Participation

The survey period spanned from mid-June through August 2019. The diabetes association mailed a paper-based questionnaire and a return envelope to all eligible members (*N* = 364). Members were invited to participate in the study via the associations’ newspaper. We collected 109 questionnaires, 100 of them were eligible for analysis after application of the half item criterion. The response rate was 29.9%. Participation in the survey was completely voluntary, and informed consent was given by completing the questionnaire and mailing it back to the association.

### 2.3. Instrument

The questionnaire prepared for the survey consisted of five parts. The first part began by asking how often participants had interacted with health care workers in four health professions (i.e., general practitioners, nurses and dieticians in outpatient care, nurses and diabetologists at diabetes centers in hospitals and dieticians at hospitals) in the last year. It also contained a seven-item question asking participants to rate aspects of their communication and interaction with the indicated professions on a 4-point Likert scale ranging from 1 (*never*) to 4 (*often*). In particular, the seven items asked participants about the time dedicated to the encounter, the possibility for them to ask questions, their sense of being listened to and understood by the health professionals, their agreement with the professionals about which changes to pursue, and to what degree nutritional and physical activity habits were discussed. The first part of the questionnaire was additionally prepared with reference to the results of a qualitative study [33].

Next, the second and third parts consisted of two scales [29,32], each validated in Italian and containing 18 items seeking responses on a Likert scale ranging from 1 (*not at all true*) to 7 (*completely true*) and six visual analog scale items assessing nutritional and physical activity habits. In the fourth part, we used Ishikawa et al.’s [34,35] health literacy scale validated in German, albeit reduced to nine items, to assess the communicative and critical health literacy of patients with DM2. That and all other scales were included in the questionnaire with the authors’ permission. Because the scales in the second and third parts were unavailable in German and the scale in the fourth part was unavailable in Italian, we had the scales translated following Wild et al.’s [36] process—that is, sent each scale to three independent bilingual experts in the field and had two experts compare their translations until consensus was reached. None of those experts were otherwise involved in the study.

Last, the fifth part of the questionnaire asked participants about their clinical status and experience with DM2, the results of which appear in Table 1a,b. We also conducted a pretest with 10 individuals with DM2 and interviewed two of them about their difficulties, if any, with understanding questions in a cognitive debriefing.

### 2.4. Statistical Analysis

In our cross-sectional pilot study, descriptive statistics for each variable included in data collection were calculated to analyze the participants’ characteristics. Herein, each stage of change and psychological process was calculated according to Centis et al. [29]. This meant that each of the five stages of change and each single psychological process has been obtained from a combination of the value expressed by the participant to specific questions in part 2 and part 3 of the questionnaire. All values are expressed in a scale ranging from 0 (not at all) to 100 (completely). With this value we indicated the range of the score for each behavioral domain and we meant all 5 stages of change as we have explained throughout the manuscript. For quantitative variables, the main indicators of centrality and variability were calculated, while the association between categorical variables was investigated with Pearson’s chi-square test and Fisher’s exact test. The Mann–Whitney test and Kruskal–Wallis test were also used to evaluate between-group differences after the non-normality distribution of the variables examined in the analysis was verified. Considering the high number of statistical tests performed, the alpha level of each difference was adjusted downward following Benjamini and Hochberg’s [37] procedure. In another statistical analysis concerning nutritional and physical activity habits, we aggregated the five stages of change into two stages, which we considered the “passive stage” (i.e., the precontemplation and contemplation stages) and the “active stage” (i.e., the preparation, action, and maintenance stages). That aggregation was done with reference to Centis et al. [29]. To identify which variables were significantly associated with being in the active stage, we analyzed the data with a binary logistic regression model following Wald’s backstep method, which allows analyzing the asymmetrical relationship (i.e., dependence) of a variable of interest (i.e., dependent binary variable) and one or more variables (i.e., independent variables). We performed all analyses with IBM’s SPSS statistical software (v24, IBM, Armonk, NY, USA) and considered a *p* value less than 0.05 (i.e., two-tailed test) to indicate statistical significance.

## 3. Results

Findings for participants’ sociodemographic variables and frequency of contact with the health professions appear in Table 1a, whereas their clinical characteristics appear in Table 1b. Our sample contained more males than females, most participants were at least 70 years old, and most had had DM2 for more than 10 years. Participants generally indicated regularly taking their prescribed medication and measuring their blood glucose. A great proportion of them was not insulin dependent, they were on treatment for cholesterol and blood pressure but did not have any complications of the disease nor had they a cardiovascular disease. Not all participants had had contact with all four health professions, however, and only nearly half of them had participated once in a training course. The health literacy level of participants was mostly low, and the BMI was distributed nearly at one third from overweight, normal and obese.

**Figure 1:**
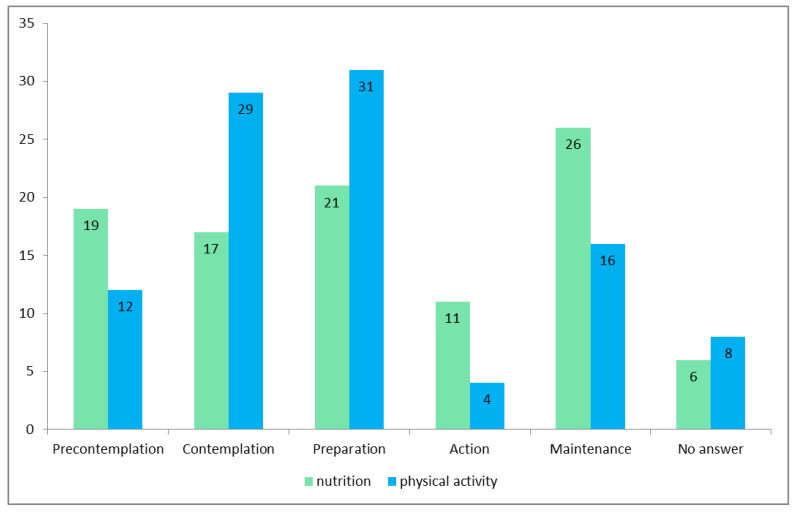
Percentage distribution according to the stage of change.

The participants were distributed among all five stages of change. Figure 1 shows the distribution of participants (%) in the 5 different stages (green) for nutritional habits and (blue) for physical activity. Regarding nutritional habits, 26% of participants were in the maintenance stage, followed by 21% in the preparation stage, 19% in the precontemplation stage and 17% in the contemplation stage. The lowest percentage for nutritional (11%) and physical activity habits (4%) was for participants in the action stage. By contrast, for physical activity habits, 31% of participants were in the preparation stage, 29% in the contemplation stage, 16% in the maintenance stage and 12% in the precontemplation stage.

Table 2 displays the mean scores for nutritional and physical activity habits for all five stages of change and the six psychological processes among participants in our sample. With this we make transparent how “high” it was in the single stage of change or psychological process and for what domain of habits. The *p* values refer to the difference between the mean scores of nutritional and physical activity habits referring to each stage of change and psychological process.

As shown in Table 2, significant differences surfaced between the two studied habits for all stages of change except contemplation. Regarding the psychological processes, significant differences emerged for temptation and readiness for change.

All stages of change and psychological processes listed in Table 2 were further analyzed to identify any associations with clinical and sociodemographic data, as well as communication- and interaction-related variables, with the three health professions most often visited in the past year: general practitioners, nurses and diabetologists at diabetes centers in hospitals. Because our analysis assessed which variables could influence the participants’ stages of change and psychological processes, it included only participants who reported having had at least one interaction with those three health professionals in the past year.

Table 3a,b present the results for significantly associated variables concerning nutritional habits, whereas significantly associated variables concerning habits of physical activity appear in Table 4a,b. Among the notable results and as an example of how to read the results presented in the tables for participants in the precontemplation stage, the higher mean score obtained for males (52.2), as reported in Table 3a, differed significantly *(p* = 0.018) from that observed for females (38.0). This interpretation was adopted for all mean scores reported (in brackets) after the variable in the middle row of Table 3a,b and Table 4a,b, because we had to compare five stages of change and six psychological processes with 27 different clinical and sociodemographic or communication and interaction variables (297 comparisons). To warrant the readability of the information given in those tables we therefore always report the higher mean score obtained for the referred variable with the modality displayed in brackets.

As shown in Table 3a, values of preparation-and action stages were significantly higher in participants having time since diagnosis for DM2 shorter than 10 years, than in those with a longer diagnosis period. Concerning the maintenance stage, its value was higher among participants that were declared to be well informed about their disease. Values in weighing the pros and cons of change were significantly higher in participants with a HbA1c level (≥7.5%), while the presence of complications was associated with higher temptation values.

In Table 3b, results for all significant associations emerged between the indicated stage of change or psychological process and for which of the indicated communication and interaction variables regarding the nutritional habits are shown. e.g., the higher mean score for participants having temptations regarding their nutritional habits indicated that there was nearly never enough time in the encounter with the nurse in hospital (75.6) differing significantly (*p* = 0.012) from those indicating that they had enough time in the encounter.

Table 4a displays the results for significant associations between stages of change or psychological processes and clinical or sociodemographic variables for the domain of physical activity habits. To facilitate the readers’ interpretation, we give the following example: participants in the contemplation stage having a low health literacy (mean = 54.6) differed significantly (*p* = 0.036) from those with a high health literacy.

As is evident from Table 4a, several stages of change and psychological processes were associated significantly with the waist to height ratio: preparation stage, readiness for change and stability of change. Except for a discrepancy, we observed higher values in correspondence to low risk.

In Table 4b, the obtained significant associations between stages of change or psychological processes and communication and interaction variables for the domain of physical activity habits are presented. That means, for example, that participants weighing their pros and cons (discrepancy) of changing physical activity habits indicated that they rarely felt being understood by their general practitioner (mean = 54.8). This differed significantly (*p* = 0.012) from those who felt being understood by them.

Looking at Table 4b, the affirmation “I feel that he or she understands me” appears frequently between the variables associated with stages of change or psychological processes. Particularly, participants that expressed difficulties in feeling understood with GP indicated higher values concerning discrepancy and self-efficacy. On the opposite, higher values of precontemplation were observed among participants which felt understood by nurses.

Table 3a,b and Table 4a,b display the significant associations that emerged between stages of change, psychological processes, and other variables. As shown, more associations with clinical variables surfaced for physical activity habits than for nutritional habits. Beyond that, associations for six of seven variables related to communication items were significant for both types of habits. The stage predominantly associated with those variables was precontemplation. Of them, six variables were significantly associated with nurses and diabetologists in the diabetes centers of hospitals regarding discussing the habits indicated, the possibility of asking questions, and the feeling of being listened to and understood by the professionals. Higher mean scores at that stage were observed for participants who had had more frequent contact with the professionals. For participants in the maintenance stage, both types of habits were significantly associated with high health literacy, whereas it was low for physical activity habits for participants in the contemplation stage. Last, for participants in the preparation stage, an association emerged with the absence of cardiovascular disease for both types of habits.

More frequent interaction with the health professionals seemed to increase supportive psychological processes such as self-efficacy and stability of change. By contrast, for challenging processes such as discrepancy and temptation, values were higher among participants with less frequent interaction and communication with the professionals, as indicated in Table 3b Table 4b. Participants with normal HbA1c levels rated their physical activity habits as having greater importance than those with non-normal HbA1c levels. Furthermore, waist-to-height ratio (WtHR) was not associated with nutritional habits, whereas for physical activity habits it was inversely correlated with higher importance, self-efficacy, readiness for change, and stability of change. A higher mean score for discrepancy was expressed by participants with higher WtHR.

By extension, following Centis et al. [29], we dichotomized the five stages into two new ones, labeled “passive stage” (=precontemplation-and contemplation stage) and “active stage”, (=preparation-, action-and maintenance stage) and associated them with the six psychological processes as part of the scale and the primary sociodemographic and clinical variables. We analyzed the seven items asked in the first part of the questionnaire concerning communication and interaction with general practitioners, nurses and diabetologists at the diabetes center. Table 5 reports the mean percentage scores calculated for the two new stages.

Participants in the passive stage of nutritional and physical activity habits had a significantly higher mean score for discrepancy and temptation than respondents in the active stage. At the same time, for importance, self-efficacy and stability of change, participants in the active stage of both types of habits had a significantly higher mean score for readiness for change but only in terms of physical activity habits.

A significant association also emerged between the new aggregated stages (i.e., passive and active) and health literacy for both types of habits, HbA1c level with nutritional habits and WtHR with physical activity habits. Participants in the active stage rated themselves as having higher health literacy (28.9% for nutritional habits, *p* = 0.006; 32% for physical activity habits, *p* = 0.030) than ones in the passive stage (5.7% for nutritional habits, *p* = 0.006; 12.5% for physical activity habits, *p* = 0.030). Likewise, HbA1c level among participants in the active stage was in 78.9% normal compared with 50% among participants in the passive stage (*p* = 0.016). Participants in the passive stage more frequently reported a higher WtHR than ones in the active stage (70.8% vs. 34.2%, *p* = 0.005).

To identify which variables were significantly associated with being in the active stage, we conducted a logistic regression analysis with discrepancy, importance, self-efficacy, temptation, readiness for change and stability of change as psychological processes; health literacy (i.e., “Low” and “High”) as an indicator for handling information; HbA1c level (i.e., “Normal” and ”Not normal”), compulsory school degree or higher and high cholesterol as input variables; and the aggregated stages of change, passive or active, as dependent variables. All variables or items referring to communication or interaction and WtHR were excluded, due to the limited availability of data for the analysis.

In the final model, we observed associations with being in the active stage concerning nutritional habits (*n* = 66) for low cognitive dissonance, expressed as discrepancy (odds ratio (OR) = 0.954; 95% confidence interval (CI) = 0.913–0.997, *p* = 0.036), the condition of being self-effective (OR = 1.124, 95% CI = 1.046–1.209, *p* = 0.001), and normal HbA1c level (OR = 0.157, 95% CI = 0.028–0.885, *p* = 0.036).

Similarly, physical activity habits (*n* = 70) in the active stage were associated with low discrepancy (OR = 0.958, 95% CI = 0.933–0.984, *p* = 0.002), high self-efficacy (OR = 1.047, 95% CI = 1.012–1.084, *p* = 0.008), and high cholesterol (OR = 5.123, 95% CI = 1.223–21.465, *p* = 0.025).

In sum, the most important results of the pilot study were that people at a higher stage of change seemed to:

- Use more supportive psychological processes such as self-efficacy;
- Have higher health literacy;
- Have better clinical status for both habits.

## 4. Discussion

The TTM regarding nutritional or physical activity habits has been used to study weight loss, vegetable and fruit consumption, fat intake, and self-care habits among individuals with DM2 [19,24,25,26,28,39]. The model has also been applied to examine multiple behaviors in different domains, including health promotion and preventive medicine [18,39], often in studies with cross-sectional designs. Other studies, by contrast, have focused on investing predictors for successful dietary changes and how people with different intentions in adopting regular physical activity habits have used processes of change, self-efficacy and decisional balance [19,20,24]. Kirk et al. [25], for example, revealed the connectedness of habitual physical activity and different constructs of the TTM in people with DM2 and cardiovascular disease. In Ataee et al.’s [27] study, an association between level of education and physical activity was observed, while Guicciardi et al. [26], similar to us, did not measure processes of change but nevertheless found a correlation between people with DM2 being at a higher stage and being more active. In Centis et al.’s [29] multicenter, cross-sectional study, which helped to guide our research, the authors used Spiller et al.’s [32] scale to evaluate the five stages of change and six psychological processes for both kinds of habits.

To evaluate the disposition of people with DM2 regarding changes in nutritional and physical activity habits and identify factors associated with them, we combined seven items concerning communication and interaction with three health professions and the health literacy scale developed by Isikawa [34] to assess how such individuals process information. As mentioned in the paper’s introduction, no comparable study integrating patient–professional interaction and behavior change in cases of chronic disease was found in PubMed or CINAHL. An exception is Nagaraju’s [28] work, however, which was published after we had completed our study. Nevertheless, we believe that we have built upon their findings about how situational self-efficacy can be improved by intensified communication about changing personal behavior. With our pilot study, we revealed findings regarding the importance of communicating and interacting with different health professions and that not all such professions seem to be included in the process of caring for the specific group of patients with DM2. As reported in the results, in different stages of change the importance of communication variables seemed to differ for participants. Among ones classified in the precontemplation stage, we observed a more frequent need to interact with nurses and diabetologists at hospitals by allowing patients to ask questions, be listened to, feel understood, and even talk about nutritional and physical activity habits. Since individuals in the precontemplation stage are typically unaware that they have any behavioral problems, the desire of simply talk with health professionals seems characteristic of that stage. We thus propose that communication can help to illuminate the need for behavior change.

Added to that, participants in the contemplation stage—that is, when people realize that they have a problematic behavior—reported that the time, if any, dedicated to their nutritional habits during meetings with nurses at hospitals was often too brief. Furthermore, concerning the feeling of being understood, which is central to patients’ discrepancy in weighing the advantages and disadvantages of changing, they reported rarely feeling understood by general practitioners regarding both kinds of habits studied. All of those findings can be interpreted to corroborate Prochaska et al.’s [21] recommendation to “do the right things (processes) at the right time (stages)”.

Meanwhile, self-efficient participants reported often feeling understood by their general practitioner and that nurses often listened to them carefully. As expected, participants experiencing temptation reported that they rarely discussed their physical activity habits with general practitioners, because they and nurses rarely had enough time during each meeting. Participants with stable changes in their nutritional habits also reported often feeling understood by their general practitioners. Against our expectations, however, self-efficient participants expressed seldom feeling understood by their general practitioners, which could mean that rarely interacting with physicians who do not support behavioral changes is better than regularly being disappointed by them. That result may also undergird the association of self-efficacy and rarely agreeing with the decisions of general practitioners during the process of changing physical activity habits.

Participants in the contemplation stage for physical activity habits reported lower health literacy, meaning that they had less skill in connecting information to their personal health situations. However, participants in the maintenance stage expressed higher health literacy for both types of habits studied, which could indicate the need for health professionals to consider health literacy more systematically and according to stages in the meetings with patients.

Whereas other authors [29] have reported an association of body mass index (BMI) with the five stages, we detected a connection only for the precontemplation stage (i.e., participants who were obese) and self-efficacy (i.e., participants with normal weight). We calculated the WtHR, an indicator of the need for weight loss according to Ashwell et al. [38], because it seemed to be more specific than BMI alone. All associations of WtHR found in our study were for physical activity habits, and we could classify participants as low or high risk but never as no risk, probably due to the relatively old age and long disease duration among participants. All associations with low risk were obtained for participants in the preparation stage, who likewise had high levels of self-efficacy, importance, readiness for change, and stability of change. By contrast, patients’ higher discrepancy was associated with being at higher risk—that is, having a greater need to lose weight.

As described earlier, the psychological processes can be used to guide external assistance and self-help. In our analysis of the active and passive stages, however, no significant associations surfaced for readiness for change regarding nutritional habits, possibly due to the greater preparedness of participants in our sample, who could have already changed their habits and therefore saw no additional need to do so again.

People with DM2 are often considered to represent an especially difficult patient group due to the non-urgency of their need for lifestyle changes, and the findings of the logistic regression analysis revealed some interesting insights into that topic. For example, participants with high cholesterol were associated with being in the active stage regarding their physical activity habits, in which case the driver could be anxiety about experiencing a more severe problem (e.g., stroke).

## 5. Strength and Limitations

Using consistent scales when available, our lengthy questionnaire captured detailed information about how the perceptions of individuals with DM2 can drive changes in health care delivery.

The variables related to communication were identified in a previous qualitative explorative interview study with participants with DM2 in an outpatient care setting. For our study, we mixed classic issues in communication with a concrete evaluation of services (e.g., whether health professionals and patients talked about nutritional and physical activity habits). Among the limitations, the convenience sample reflected the distribution of age but not of sex (i.e., 57% males vs. 48% females) in the population studied. Beyond that, the limited number of participants in our sample was another clear limitation.

Last, behavior change could be more thoroughly studied over longer periods and not in cross-sectional studies.

## 6. Conclusions

The findings of our pilot study underscore the importance of communicating about nutritional habits and physical activity habits as well as interacting with different health professionals. We found that not all health professionals seem to be included in the process of caring for this specific patient group. Additionally, as health professionals may not consistently consider the patient’s individual, social and psychological status, the integration of stages and processes of change can improve diabetes care. The combination of the stages of change and how they relate to psychological processes can afford meaningful insights into the potential of the internal and external communication capabilities of health professionals and should be examined as possible elements for a patient evaluation model.

## Figures and Tables

**Table 1:** (**a**) Descriptive characteristics of participants and their frequency of contact with health professions in the last year (*N* = 100). (**b**) Clinical characteristics of participants (*N* = 100).

(**a**)	
**Variable**	**Frequency (%)**
***Sex***	
Male	57
Female	40
Missing	3
***Age group (in years)***	
<64	13
65–69	20
≥70	65
Missing	2
***Level of education*^1^**	
Compulsory school degree	53
Secondary or higher degree	47
***Self-evaluation of economic status***	
Insufficient	6
Average	78
Good	16
***Occupational status***	
Active	13
Retired	87
***Cohabitation status***	
Living alone	16
Living with someone	78
Missing	6
**Frequency of contact with health the following professions ^2^**	
***General practitioner***	
Never	27
1 or 2 times per year	42
3 or more times per year	30
Missing	1
***Nurse in outpatient care***	
Never	66
1 or 2 times per year	21
3 or more times per year	9
Missing	4
***Nurse at hospital***	
Never	23
1 or 2 times per year	59
3 or more times per year	16
Missing	2
***Dietician in outpatient care***	
Never	82
1 or 2 times per year	10
3 or more times per year	5
Missing	3
***Dietician at hospital***	
Never	78
1 or 2 times per year	8
3 or more times per year	12
Missing	4
***Diabetologist at hospital***	
Never	16
1 or 2 times per year	61
3 or more times per year	20
Missing	3
(**b**)	
**Variable**	
***Health literacy*^3^**	
Low	76
High	22
Missing	2
*Body mass index class* *	
Normal	31
Overweight	40
Obese	26
Missing	3
*Weight-to-height ratio* *^,4^	
<0.5	0
0.5–0.6	34
>0.6	34
Missing	32
***Duration of diabetes***	
<5 years	11
5–10 years	11
>10 years	78
***Insulin dependence?***	
Yes	42
No	57
Missing	1
***Regular self-measurement of blood glucose?***	
Yes	93
No	6
***HbA1c level*^5^**	
Normal (<7.5)	51
Not normal (≥7.5)	22
Missing	27
***Prescribed and taking medication?***	
Yes	91
No	7
Missing	2
***Presence of complications?***	
Yes	32
No	64
Missing	4
***Treatment for high blood pressure?*^6,7^**	
Yes	65
No	34
Missing	1
***Treatment for high cholesterol?*^7^**	
Yes	62
No	36
Missing	2
***Coronary heart disease?***	
Yes	23
No	75
Missing	2
***Participating in individual or group training*** *?*	
Yes	48
No	52

^1^ Level of education was classified into compulsory school degree (i.e., primary or secondary school degree) and higher education (i.e., vocational degree, high school diploma, or university degree). ^2^ Frequency of contact with the health professions was aggregated to “1 or 2 times per year” and “3 or more times per year.” * Values were calculated from data collected (i.e., weight in kg, height in cm, and waist circumference in cm)**.** ^3^ High health literacy indicates scores greater than or equal to the median on at least seven of nine items with the max. value of 4. ^4^ Waist-to-height ratio was calculated as the proportion of waist circumference to height, according to Ashwell’s [38] method. ^5^ The given HbA1c levels presented were classified as “Normal” (i.e., <7.5%) and “Not normal” (i.e., ≥7.5%)**.** ^6^ Data for high blood pressure refer to the last 5 years. ^7^ “High” blood pressure and “high” cholesterol treatment were defined according to indications of high blood pressure and cholesterol as well as according to drug therapy.

**Table 2:** Mean (*M*) scores and standard deviation (*SD*) of stages of change and of psychological processes for nutritional and physical activity habits.

Stage of Change	Nutritional Habits		Physical Activity Habits		*p*
	*M*	*SD*	*M*	*SD*	
Precontemplation	47.6	±25.0	27.3	±24.9	<0.000
Contemplation	51.7	±25.3	50.3	±27.5	0.650
Preparation	53.3	±28.8	62.4	±28.2	0.030
Action	52.8	±25.9	42.0	±31.1	0.009
Maintenance	67.3	±22.6	58.8	±31.2	0.011
Psychological process					
Discrepancy	38.6	±23.0	42.5	±25.7	0.134
Importance	75.9	±17.0	74.8	±17.6	0.613
Self-efficacy	63.7	±21.1	61.8	±21.7	0.398
Temptation	51.1	±28.1	33.0	±23.8	<0.000
Readiness for change	48.2	±28.6	63.8	±23.7	<0.000
Stability of change	61.2	±24.3	58.7	±25.4	0.398

Green for nutritional habits and blue for physical activity.

**Table 3:** (**a**) Stages of change or psychological processes and clinical or sociodemographic variables showing significantly different mean scores in the domain of nutritional habits. (**b**) Stages of change or psychological processes and communication or interaction variables showing significantly different mean scores in the domain of nutritional habits.

(**a**)		
**Stage of Change or Psychological Process**	**Clinical or Sociodemographic Variable** (Higher Mean Score of Stage of Change and Psychological Process)	***p***
**Precontemplation**	**Sex (Male = 52.2)**	0.018
	**HbA1c level**^1^ (Not normal = 55.2)	0.036
**Contemplation**	**Cardiovascular disease**^2^ (No = 55.3)	0.036
	**Attendance of a training course**^2^ (No = 58.29)	0.018
**Preparation**	**Cardiovascular disease**^2^ (No = 56.0)	0.018
	**Time since diagnosis**^3^ (<10 years = 63.5)	0.036
**Action**	**Time since diagnosis**^3^ (<10 years = 65.8)	0.018
**Maintenance**	**Health literacy level**^4^ (Well informed = 80.2)	0.018
**Discrepancy**	**Sex** (Female = 44.2)	0.018
	**HbA1c level**^1^ (Not normal = 49.4)	0.036
**Temptation**	**Complications**^2^ (Yes = 58.8)	0.018
**Readiness for change**	**Sex** (Female = 56.7)	0.018
(**b**)		
	**Communication or Interaction Variable** (Higher Mean Score of Stage of Change and Psychological Process)	***p***
**Precontemplation**	**We talk about my nutritional habits**^5^ (DIAB) (Sometimes/Often = 51.1)	0.012
	**We talk about my physical activity habits**^5^ (DIAB) (Sometimes/Often = 55.6)	0.024
	**We talk about my physical activity habits**^5^ (NH) (Sometimes/Often = 53.8)	0.036
**Contemplation**	**There is enough time for each meeting**^5^ (NH) (Never/Rarely = 76.2)	0.012
**Preparation**	**We decide together what changes I will undertake**^5^ (NH) ^1^(Sometimes/Often = 61.3)	0.012
**Maintenance**	**I feel that he or she understands me**^5^ (NH) (Sometimes/Often = 71.3)	0.012
	**There is enough time for each meeting**^5^ (NH) (Sometimes/Often = 71.7)	0.024
**Discrepancy**	**I feel that he or she understands me**^5^ (GP) (Never/Rarely = 52.6)	0.012
**Self-efficacy**	**I feel that he or she understands me**^5^ (GP) ^1^ (Sometimes/Often = 66.3)	0.012
	**He or she listens to me carefully**^5^ (NH) (Sometimes/Often = 67.4)	0.024
**Temptation**	**There is enough time for each meeting**^5^ (NH) (Never/Rarely = 75.6)	0.012
	**We talk about my physical activity habits**^5^ (GP) (Never/Rarely = 57.6)	0.024
**Stability of change**	**I feel that he or she understands me**^5^ (GP) (Sometimes/Often = 64.7)	0.012

Note: ^1^ HbA1c level was dichotomized as “Normal” (<7.5%) and “Not normal” (≥7.5%). ^2^ CDC, complications, and attendance of training course were dichotomized as “Yes” and “No.” ^3^ Diagnosis was dichotomized as “<10 years” and “≥10 years.” ^4^ Health literacy level was dichotomized as “Low” and “High” (i.e., intended as well informed vs. less informed). ^5^ Communication variables for the indicated health profession were dichotomized as “Never/Rarely” and “Sometimes/Often”. GP = general practitioner, NH = nurse at hospital, DIAB = diabetologist at hospital. Green for nutritional habits.

**Table 4:** (**a**) Stages of change or psychological processes and clinical or sociodemographic variables showing significantly different mean scores in the domain of physical activity habits. (**b**) Stages of change or psychological processes and communication or interaction variables showing significantly different mean scores in the domain of physical activity habits.

(**a**)		
**Stage of Change or Psychological Process**	**Clinical or Sociodemographic Variable** (Higher Mean Score of Stage of Change or Psychological Process)	***p***
**Precontemplation**	**Sex** (Male = 32.5)	0.018
	**BMI class**^1^ (Obese = 37.9)	0.036
**Contemplation**	**Self-evaluation of economic status**^2^ (Insufficient = 59.7)	0.018
	**HL level**^3^ (Poorly informed = 54.6)	0.036
**Preparation**	**BMI class**^1^ (Normal = 69.3)	0.018
	**WtHR**^4^ (Low risk = 73.4)	0.036
**Maintenance**	**HL level**^3^ (Well informed = 72.6)	0.018
**Discrepancy**	**Self-evaluation of economic status**^2^ (Insufficient = 65.0)	0.018
	**WtHR**^4^ (High risk = 49.4)	0.036
**Importance**	**WtHR**^4^ (Low risk = 83.1)	0.018
	**HbA1c level**^5^ (Normal = 78.7)	0.036
**Self-efficacy**	**WtHR**^4^ (Low risk = 70.3)	0.018
	**BMI class**^1^ (Normal = 71.0)	0.036
**Temptation**	**Age class**^6^ (<70 years = 43.0)	0.018
**Readiness for change**	**WtHR**^4^ (Low risk = 72.0)	0.018
**Stability of change**	**WtHR**^4^ (Low risk = 68.6)	0.018
(b)		
	Communication or Interaction Variable (Higher Mean Score of Stage of Change or Psychological Process)	*p*
**Precontemplation**	**He or she allows me to ask questions** ^7^ **(NH) (Sometimes/Often = 28.6)**	0.012
	**He or she listens to me carefully**^7^ (NH) (Sometimes/Often = 27.2)	0.024
	**I feel that he or she understands me**^7^ (NH) (Sometimes/Often = 26.6)	0.036
	**We decide together what changes I will undertake**^7^**(GP)** (Sometimes/Often = 31.2)	0.048
**Discrepancy**	**I feel that he or she understands me**^7^ (GP) (Never/Rarely = 54.8)	0.012
**Self-efficacy**	**I feel that he or she understands me**^7^ (GP) (Never/Rarely = 72.5)	0.012
	**We decide together what changes I will undertake**^7^ (GP) (Never/Rarely = 68.2)	0.024
**Temptation**	**We talk about my physical activity habits**^7^ (GP) (Never/Rarely = 40.4)	0.012

Note: WtHR = waist-to-height ratio; BMI = body mass index; HL = health literacy. ^1^ BMI was classified as “Normal,” “Overweight,” and “Obese.” ^2^ Self-evaluated economic status was classified as “Insufficient,” “Average,” and “Good.” ^3^ Health literacy level was dichotomized as “Low” and “High” (i.e., intended as well informed vs. less informed). ^4^ WtHR was dichotomized as “Low risk” and “High risk.” ^5^ HbA1c level was dichotomized as “Normal” (<7.5%) and “Not normal” (≥7.5%). ^6^ Aggregated age class, in years, was dichotomized as “<70” and “≥70.”. ^7^ Communication variables for the indicated health profession were dichotomized as “Never/Rarely” and “Sometimes/Often”. GP = general practitioner, NH = nurse at hospital, DIAB = diabetologist at hospital. Blue for physical activity.

**Table 5:** Mean scores of aggregated stages of change for psychological processes according to nutritional and physical activity habits.

Psychological Processes	Nutritional Habits			Physical Activity Habits		
	Passive Stage	Active Stage	*p*	Passive Stage	Active Stage	*p*
Discrepancy	49.2	31.9	<0.000	56.9	31.0	<0.000
Importance	67.8	81.3	<0.000	68.3	80.2	0.001
Self-efficacy	48.8	73.0	<0.000	52.2	70.5	<0.000
Temptation	61.8	42.3	0.002	43.4	26.1	<0.000
Readiness for change	44.3	51.6	0.145	54.3	71.9	<0.000
Stability of change	47.2	70.2	<0.000	45.5	69.4	<0.000

Green for nutritional habits and blue for physical activity.
